# A Double-Layer LSTM Model Based on Driving Style and Adaptive Grid for Intention-Trajectory Prediction

**DOI:** 10.3390/s25072059

**Published:** 2025-03-26

**Authors:** Yikun Fan, Wei Zhang, Wenting Zhang, Dejin Zhang, Li He

**Affiliations:** 1 College of Electronics and Information Engineering, Shenzhen University, Shenzhen 518000, China; 2200432090@email.szu.edu.cn (Y.F.); 2210433081@email.szu.edu.cn (W.Z.); 2 Institute of Applied Artificial Intelligence of the Guangdong-Hong Kong-Macao Greater Bay Area, Shenzhen Polytechnic University, Shenzhen 518000, China; z20210145@szpu.edu.cn; 3 School of Architecture & Urban Planning, Shenzhen University, Shenzhen 518000, China; djzhang@szu.edu.cn; 4 College of Mechatronics and Control Engineering, Shenzhen University, Shenzhen 518000, China

**Keywords:** trajectory prediction, LSTM, driving style, grids in interaction, autonomous vehicle

## Abstract

In the evolution of autonomous vehicles (AVs), ensuring safety is of the utmost significance. Precise trajectory prediction is indispensable for augmenting vehicle safety and system performance in intricate environments. This study introduces a novel double-layer long short-term memory (LSTM) model to surmount the limitations of conventional prediction methods, which frequently overlook predicted vehicle behavior and interactions. By incorporating driving-style category values and an improved adaptive grid generation method, this model achieves more accurate predictions of vehicle intentions and trajectories. The proposed approach fuses multi-sensor data collected by perception modules to extract vehicle trajectories. By leveraging historical trajectory coordinates and driving style, and by dynamically adjusting grid sizes according to vehicle dimensions and lane markings, this method significantly enhances the representation of vehicle motion features and interactions. The double-layer LSTM module, in conjunction with convolutional layers and a max-pooling layer, effectively extracts temporal and spatial features. Experiments conducted using the Next Generation Simulation (NGSIM) US-101 and I-80 datasets reveal that the proposed model outperforms existing benchmarks, with higher intention accuracy and lower root mean square error (RMSE) over 5 s. The impact of varying sliding window lengths and grid sizes is examined, thereby verifying the model’s stability and effectiveness.

## 1. Introduction

The advancement of autonomous vehicles is fundamentally anchored in ensuring safety [1], which is a critical objective that requires overcoming pivotal challenges in terms of localization, perception, prediction, and path planning. These tightly coupled modules are indispensable for enhancing vehicle safety, system performance, and reliability in dynamic and complex environments. Among them, perception—serving as the cornerstone of autonomous driving systems—relies on multi-modal sensors (e.g., LiDAR, cameras, and radar) to capture real-time environmental data such as vehicle trajectories, lane markings, and agent dimensions. These sensor-derived features then feed into downstream modules, where prediction—acting as a linchpin—bridges the gap between perception and path planning [2,3]. By analyzing data from multi-sensor inputs, prediction enables real-time hazard detection and informed decision-making under uncertain conditions [4,5]. With rapid advancements in artificial intelligence (AI), innovations in data-driven trajectory prediction are poised to accelerate the widespread adoption of autonomous vehicles, ultimately transforming transportation systems.

In general, there are three categories of traditional trajectory prediction methods [6], which comprise physics-based methods, behavior-based methods, and interaction-aware methods. Physics-based methods assume that the future movement of a vehicle depends solely on its current motion state [7,8,9]. According to dynamic models and motion models, the motion of the vehicle can be calculated by a simple and efficient prediction model. Faced with uncertainty about the current state and future changes of the vehicle, physics-based methods may not predict trajectories well over a long period of time. Behavior-based motion models provide high accuracy in longer-term motion prediction by considering vehicle behavior [10,11,12,13,14]. In those models, every vehicle is regarded as a moving entity engaged in a certain behavior, and future movement is inferred based on prior behavior. Interaction-aware motion prediction methods further consider the interactions between multiple road vehicles while making predictions. Thus, a target vehicle trajectory prediction method that considers the trajectories of surrounding vehicles and their interactions is proposed [15,16,17]. The future trajectories of on-road vehicles are usually predicted based on cost functions, which can be used to describe vehicles’ relative relationships. Cost function-based methods require no training data, but different road environments need careful designing of different cost functions, causing high labor costs. However, these traditional methods struggle to fully account for vehicle interactions and individual driving style differences in complex real-world traffic scenarios, limiting their prediction accuracy.

With the rise of artificial intelligence, data-driven approach-based trajectory prediction methods have gained favor among researchers. Due to the information mining and deep representation capabilities of recurrent neural networks (RNN) when dealing with sequential problems [18], scholars have proposed many trajectory prediction methods based on recurrent neural networks. Especially inspired by pedestrian trajectory prediction [19], LSTM networks are also used to solve the vehicle trajectory prediction problem [20,21]. The trajectory output of LSTM-based methods adopts encoder-decoder architecture, and such methods have the ability to deal with arbitrary length inputs or to generate trajectories with arbitrary length [21]. Furthermore, the Social-LSTM model [19] of modeling interactions between pedestrians has also been applied to vehicle trajectory prediction [22]. Many strategies have been proposed to improve LSTM-based and Social-LSTM-based vehicle trajectory prediction methods, such as using convolutional layers to extract spatial dependencies [22,23], modeling different types of participant interaction [24], considering environmental constraints [23], and so on. In recent years, with the emergence of Transformer, many models based on it [25,26,27] have been proposed by researchers in the field of trajectory prediction.

However, these studies have not yet taken the important role of driving style in intentions and trajectory prediction into account. A driver’s long-term driving style significantly influences future vehicle trajectories through differences in acceleration, lane-changing frequency, and other behavioral characteristics [28]. Therefore, incorporating driving style into models is essential for accurate intention and trajectory prediction. Traditional driving style characterization includes two aspects [29]: vehicle motion features (such as vehicle speed, acceleration and deceleration, brake pedal pressure, steering wheel torque, etc.) and external driving scenario features (such as lane change, average headway time, etc.). Conservative drivers typically exhibit lower acceleration and fewer lane changes, while aggressive drivers show the opposite pattern, directly affecting vehicle motion trajectories and interaction modes.

This paper proposes a novel intention and trajectory prediction method based on a double-layer LSTM model. The new method improves the grid generation method in vehicle interaction and considers the impact of the target vehicle’s driving style on intention and trajectory prediction. The input data of the double-layer LSTM model contains sequences of trajectory coordinates, driving style category value, and the grid of vehicle interaction. Compared with other methods, the proposed method demonstrates superior performance in intention and trajectory prediction.

The main contributions of this paper are as follows:

1. A novel double-layer LSTM model that integrates driving style and the grid of vehicle interaction is proposed for predicting target vehicle intentions and trajectory, which is superior to existing benchmarks;
2. A new driving style classification method based on the inverse cruise ratio is proposed to improve the accuracy of intention prediction, and its effectiveness is verified in experiments;
3. This paper proposes an adaptive grid generation strategy for vehicle interaction, and detailed analyses have been conducted in the experiments.

The organization of this paper is as follows. The Section 2 introduces the proposed method, the Section 3 describes the dataset used and the experimental results. The Section 4 summarizes the paper.

## 2. Methodology

**Figure 1:**
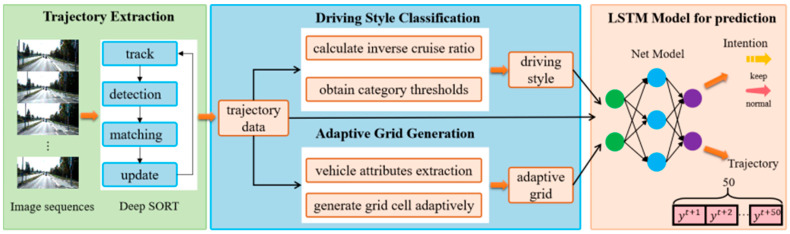
Methodology framework of the paper.

The methodology consists of four components: trajectory extraction, driving style classification, adaptive grid generation, and the LSTM model for prediction, as shown in Figure 1.

### 2.1. Trajectory Extraction

Motion trajectories play a crucial role in tracking and analyzing objects of interest, which can be extracted from video or continuous image sequences. Real-world vehicle trajectories have a significant effect on studying intention and trajectory prediction, and the trajectory data of vehicles typically include the vehicle identifier, time, two-dimensional coordinates, and vehicle dimensions. The Deep SORT algorithm [30] is widely recognized for its reliability and validity in multi-object tracking. In this work, the Deep SORT algorithm is integrated with a coordinate transformation module, which converts pixel trajectories from camera coordinates to a unified world coordinate system by incorporating sensor calibration and ego-vehicle motion compensation. This facilitates the accurate extraction of trajectory data from the multi-sensor data processed by perception modules. The trajectory data of vehicle i at the j-th time frame is denoted as $traj_{i}^{j}$:

(1) $$traj_{i}^{j}=\left[ID_{i},time^{j},\left(x_{i}^{j},y_{i}^{j}\right),\left(l_{i},w_{i}\right)\right],$$

where $ID_{i}$ is the vehicle identification number, $time^{j}$ is the j-th time frame, $\left(x_{i}^{j},y_{i}^{j}\right)$ are the *x* and *y* coordinates of the vehicle i at the j-th time frame, and $\left(l_{i},w_{i}\right)$ are the length and width of the vehicle. The trajectory data Traj is the set of all $traj_{i}^{j}$:

(2) $$Traj=\left\{traj_{i}^{j}|i\in N_{i},j\in N_{j}\right\},$$

where $N_{i}$ is the set of natural numbers, including all vehicle identification numbers, and $N_{j}$ is the set of natural numbers, including all time frames.

### 2.2. Driving Style Classification and Adaptive Grid Generation

#### 2.2.1. Driving Style Classification Based on the Inverse Cruise Ratio

Driving style largely determines driving behavior and the decision-making process, which is reflected in long-term trajectory data. Reference [31] classifies driving styles into three categories (conservative, general, and aggressive), with proportions of approximately 4:4:2. In our paper, we define the inverse cruise ratio as the classification criterion for driving styles and segment the driving style to determine the driving style category thresholds by combining the above proportion and statistical data from the NGSIM dataset [32]. In practical applications, the sliding window method is used to classify the driving style based on the calculated inverse cruise ratio and the determined thresholds.

In vehicle engineering, “cruise” refers to a vehicle traveling within a certain speed range. The cruise ratio is defined as the ratio of the time a vehicle spends in the cruise phase to its total driving time over a given segment. Based on this concept, we introduce the inverse cruise ratio, which is the ratio of the time that a vehicle spends in the braking or lane-changing phases to its total driving time over the same segment. The inverse cruise ratio is calculated as the time spent braking or changing lanes, divided by the total driving time. The calculation method is as follows:

(3) $$\tau_{ic}=\frac{\sum T_{brake}\cup \sum T_{change}}{T_{total}},$$

where $T_{brake}$ is the time when the vehicle is in the braking phase, $T_{change}$ is the time when the vehicle is in the lane-changing phase, and $T_{total}$ is the total driving time. Vehicles are considered to be braking when their average speed decreases by 20% over 3 s, and lane-changing is identified by analyzing vehicle position changes of lane over 3-s intervals. Then, we take the union of the two above as the inverse cruise time and compare it with the total time to obtain the inverse cruise ratio $\tau_{ic}$.

Braking and lane-changing behaviors are critical indicators of driving style as they reflect a driver’s responsiveness to traffic conditions and their tendency towards aggressive or conservative maneuvers. Conservative drivers typically maintain steady speeds and avoid unnecessary lane changes, resulting in lower inverse cruise ratios. In contrast, aggressive drivers frequently adjust their speeds and change lanes, leading to higher ratios. The general driving style exhibits intermediate characteristics. While driving style is a complex multifaceted concept, the inverse cruise ratio provides a concise yet effective quantification that captures essential aspects of driving behavior relevant to trajectory prediction. It focuses on key maneuvers that have significant impacts on vehicle interactions and motion patterns, making it a practical choice for classification despite the complexity of driving style. In addition, these two factors can also be obtained through the vehicle’s turn signals and brake lights in human daily driving, providing the potential for multimodal input in the future.

**Figure 2:**
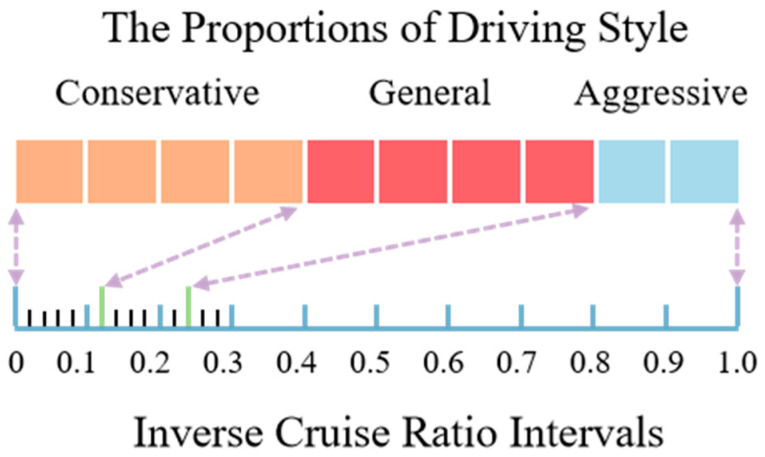
The proportion of driving style and inverse cruise ratio intervals.Arrow: the correspondence between driving style and interval boundar; blue line: a scale of 0.1; black line: a finer scale of 0.02; green line: denote boundaries for different driving styles.

According to Reference [28], driving style can be classified into three categories: conservative, general, and aggressive, with a proportion of approximately 40%, 40%, and 20%, respectively. Initially, the inverse cruise ratio of 11,779 trajectories in the six different time periods of the NGSIM dataset is calculated. Subsequently, the driving style category thresholds are obtained by segmenting and optimizing according to the proportions of the three categories, which are 0.12 and 0.24. As shown in Figure 2, the inverse cruise ratio intervals for the three categories of driving style are as follows: conservative [0, 0.12], general (0.12, 0.24], and aggressive (0.24, 1]. In practical applications, a sliding window length of the past 12 s is used to calculate the inverse cruise ratio of the driving process. Finally, according to the inverse cruise ratio and intervals mentioned above, we can classify the driving styles.

#### 2.2.2. Adaptive Grid Generation in Vehicle Interaction

Vehicle interaction influence is considered in this work to improve prediction accuracy regarding vehicle intentions and trajectories. The grid-based vehicle interaction model is constructed, which offers simplicity and validity, and an adaptive grid generation method is proposed for solving vehicle interaction problems. There are three steps involved in the new grid generation method.

**Figure 3:**
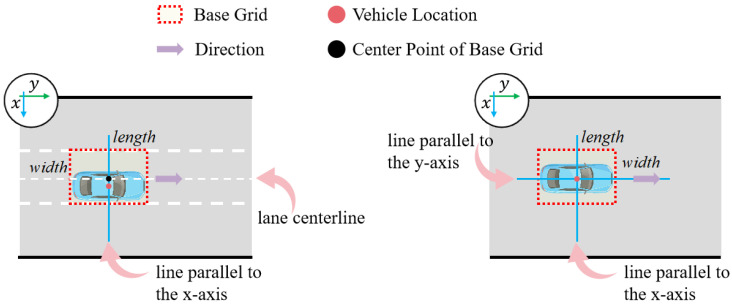
Example of a grid cell generated in two scenarios.

First, we obtain the vehicle location coordinates $\left(x,y\right)$, length l, and width w from the trajectory data. The direction of the coordinate axes is shown in the upper left corner of Figure 3.

The second step of grid cell generation is based on whether lane markings are known. When lane markings are known, the grid cell is a rectangle that has a set width indicating lane width and a set length indicating vehicle length in the adaptive strategy. The center point is the intersection of the lane centerline and the line parallel to the *x*-axis through that point $\left(x,y\right)$. The direction is parallel to the lane centerline. An example of the grid cell generated when lane markings are known is shown on the left side of Figure 3.

Otherwise, the size and center point of the grid cell will entirely depend upon the properties of the vehicle itself. The width of the grid cell is equal to twice the width of the vehicle in the adaptive strategy, while the length is the same as that of the vehicle. The center point of the base grid is at the vehicle location coordinates. The direction is parallel to the *y*-axis. An example of the grid cell generated when lane markings are unknown is shown on the right side of Figure 3.

**Figure 4:**
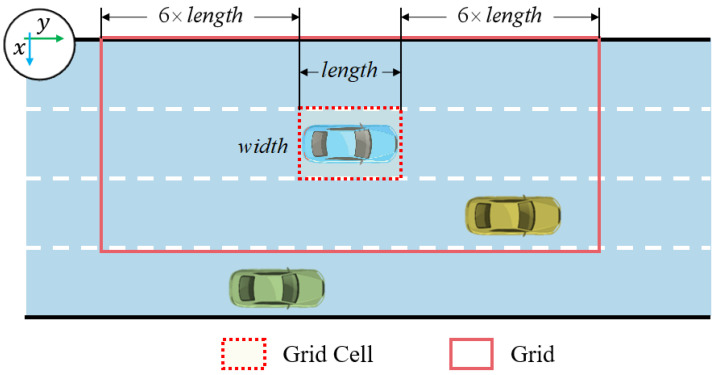
An example of a grid generated when the lane markings are known.

The third step is grid generation in vehicle interaction. The grid is generated by assuming a length of grid cell scaling of 13 and a width of grid cell scaling of 3. At the same time, the center point of the grid coincides with the center point of the grid cell and is also in the same direction. An example of a grid generated when lane markings are known is shown in Figure 4.

### 2.3. Double-Layer LSTM Model for Intention and Trajectory Prediction

**Figure 5:**
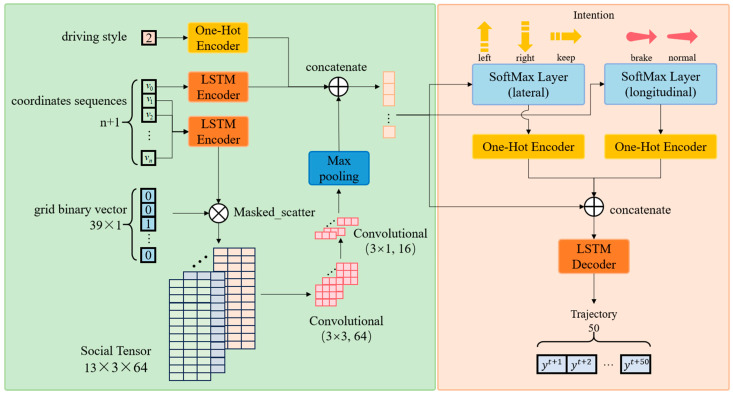
Schematic diagram of double-layer LSTM model.

The proposed double-layer LSTM model, shown in Figure 5, has an encoder and a decoder. The encoder processes input data to extract features, while the decoder generates the predicted trajectories and intentions.

The encoder includes a 32D input FC layer, a 64D LSTM encoder, a 32D motion feature FC layer, two convolutional layers, and a max-pooling layer. Input data consist of a grid binary vector (size 39), the trajectory coordinates of surrounding vehicles, and a one-hot encoded driving style vector. The encoder extracts temporal features via the LSTM layer and spatial features via convolutional and pooling layers.

The decoder includes two intention FC layers, a 128D LSTM decoder, and an output FC layer. It predicts intentions using FC layers with SoftMax activation and generates trajectories via the LSTM decoder. The output layer produces the parameters of the bivariate Gaussian distribution for the predicted vehicle’s trajectory coordinates.

#### 2.3.1. Multiple-Input Encoder and Feature Vector Concatenation

The input of the model includes a binary vector of the grid, the sequences of trajectory coordinates, and the category value of the driving style. To avoid confusion, the set of vehicles in the grid, except for the predicted vehicle, are called “surrounding vehicles” in the following section.

**Figure 6:**
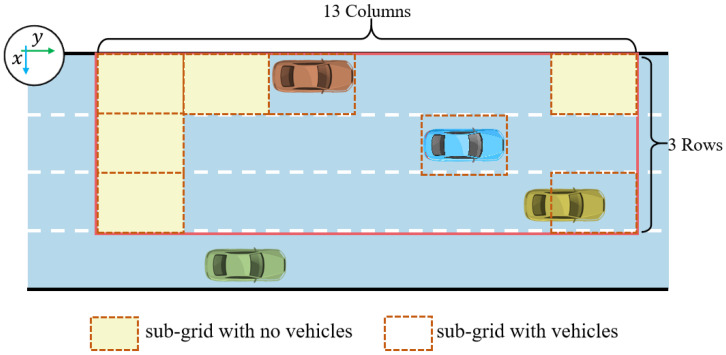
Example of the process of setting indicator vector.

**Figure 7:**
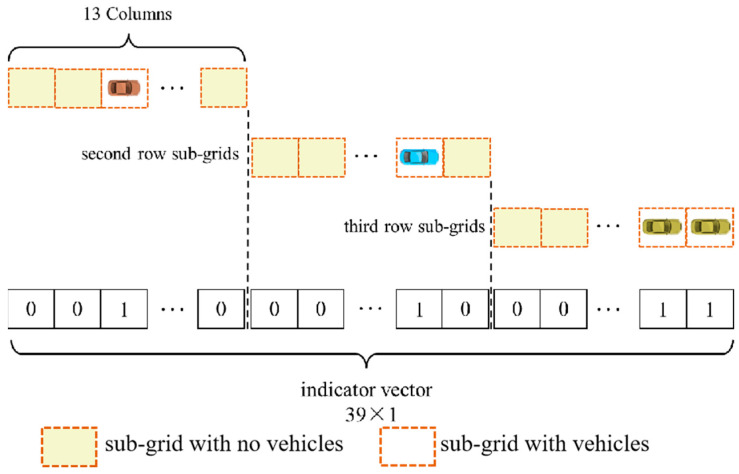
Relationship between the indicator vector index corresponding to the sub-grid and the row column number.

Here, the grid that is adaptively generated is divided into 39 sub-grids, which have 3 rows in the *x*-axis direction and 13 columns in the *y*-axis direction. The binary value of the sub-grid is set as 1 when there is a surrounding vehicle inside it; otherwise, it is set as 0. Note that the binary value of the sub-grid will be set as 0 when only the predicted vehicle is inside it. An example of the process of setting indicator vectors is shown in Figure 6. The binary values of all sub-grids are sorted into a vector, which is known as an indicator vector. The relationship between the vector index corresponding to the sub-grid and row column number is as follows:

(4) $$idx=\left(num\_row-1\right)\times 13+num\_col,$$

where idx is the vector index corresponding to the sub-grid, and num_row and num_col are the row and column numbers of the sub-grid, respectively. This is illustrated in Figure 7.

The sequences of the trajectory coordinates are denoted as X, which includes the latest $t_{h}$ trajectory coordinates of all surrounding vehicles. The surrounding vehicles are sorted in ascending order according to their sub-grid indexes:

(5) $$X=\left[v_{1},\cdots ,v_{i},\cdots ,v_{n}\right],$$

(6) $$v_{i}=\left[\left(x_{i}^{\left(t-t_{h}+1\right)},y_{i}^{\left(t-t_{h}+1\right)}\right),\cdots ,\left(x_{i}^{\left(t-1\right)},y_{i}^{\left(t-1\right)}\right),\left(x_{i}^{t},y_{i}^{t}\right)\right],$$

where n is equal to the number of surrounding vehicles, $v_{i}$ is the latest $t_{h}$ trajectory coordinates of the i-th vehicle, and $t_{h}$ is set to 30.

Then, the latest $t_{h}$ trajectory coordinates of the predicted vehicle $v_{0}$ are inserted into the beginning of the vector X. The final vector of the trajectory coordinates is expressed as X:

(7) $$X=\left[v_{0},v_{1},\cdots ,v_{i},\cdots ,v_{n}\right].$$

The LSTM encoder is utilized to learn the motion features of both the predicted vehicle and the surrounding vehicles. The sequences of the trajectory coordinates are fed through the input FC layer and the LSTM encoder, where the LSTM hidden states for each vehicle are updated frame by frame over the past $t_{h}$ frames. The final LSTM hidden states of each vehicle serve as a representation of its motion features. Subsequently, the LSTM hidden states of the predicted vehicle are passed through the motion-feature FC layer, resulting in the final acquisition of the short-term state vector of the predicted vehicle. The LSTM encoder used for each vehicle shares the same weights.

Based on the binary vector of the grid, the LSTM hidden states of all the surrounding vehicles are used to construct a social tensor performing a masked scatter operation, followed by shape adjustment. We then apply two convolutional layers and a max-pooling layer to this social tensor, adjusting its shape for subsequent concatenation, to obtain the interaction vector.

**Figure 8:**
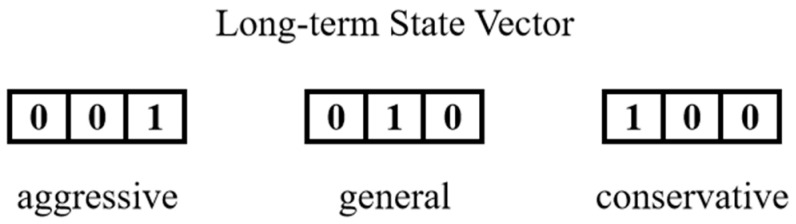
Long-term state vectors corresponding to the different driving styles.

The category value of driving style is determined by the driving style category thresholds and the calculated inverse cruise ratio. A one-hot encoder is applied to convert the category value of driving style into a binary vector, referred to as the long-term state vector of the predicted vehicle, as illustrated in Figure 8.

The short-term state vector, long-term state vector, and interaction vector are concatenated to form a 115-dimensional standardized prediction vector, which is utilized by the subsequent decoder for intention and trajectory prediction.

#### 2.3.2. LSTM Decoder for Intention and Trajectory Prediction

**Figure 9:**
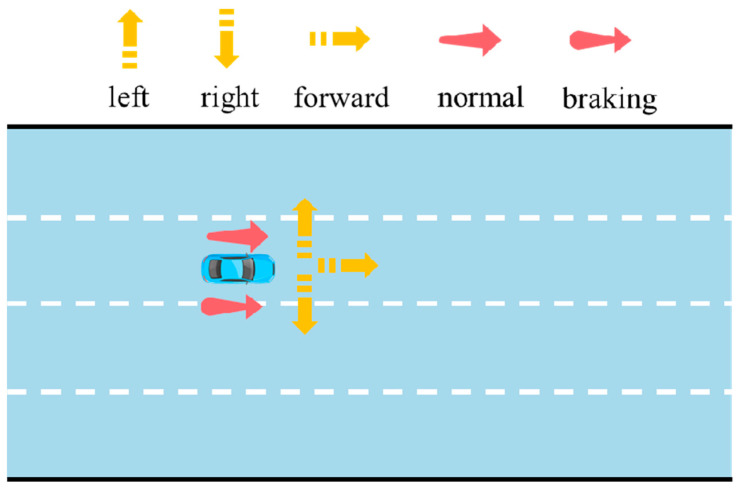
Lateral intention and longitudinal intentions.

Comprising components for intention prediction and trajectory prediction, the decoder provides the output data, including lateral intention, longitudinal intention, and predicted trajectory data. In the aforementioned inverse cruise ratio calculation process, the lateral intentions include changing to the left, changing to the right, and moving directly forward, while the longitudinal intentions encompass normal driving and braking. The identification diagram of lateral intentions and longitudinal intentions is shown in Figure 9.

The standardized prediction vector is input into two distinct intention FC layers. In the first FC layer, the dimension of the vector is reduced to 3, and it is then normalized through a SoftMax layer, resulting in a probability matrix of between 0 and 1, namely, the lateral intention probability matrix. In the second FC layer, the dimension of the vector is reduced to 2, and it is then normalized through a SoftMax layer, resulting in a probability matrix that is set between 0 and 1, namely, the longitudinal intention probability matrix. A one-hot encoder is applied to convert the intention category values with the highest intention probabilities into two binary vectors, which are, respectively, referred to as the lateral intention vector and the longitudinal intention vector.

The standardized prediction vector is concatenated with the lateral intention vector and the longitudinal intention vector to obtain a 120-dimensional standardized intention vector. This standardized intention vector serves as the hidden state input to the LSTM decoder, which updates its output frame by frame, thereby generating the predicted trajectory of $t_{f}$ frames. Subsequently, the output of the LSTM decoder, which consists of $t_{f}$ frames, is passed through the output FC layer to be reduced to five dimensions. Then, it is passed through five activation functions to respectively obtain the parameters of the Gaussian distribution for the two-dimensional coordinates.

The predicted trajectory data denoted as Y are:

(8) $$Y=\left[y^{\left(t+1\right)},\cdots ,y^{\left(t+t_{f}-1\right)},y^{\left(t+t_{f}\right)}\right],$$

(9) $$y^{\left(t\right)}=\left[\mu_{x}^{\left(t\right)},\mu_{y}^{\left(t\right)},\sigma_{x}^{\left(t\right)},\sigma_{y}^{\left(t\right)},\rho^{\left(t\right)}\right],$$

where $t_{f}$ is set at 50 and $y^{\left(t\right)}$ represents the parameters of the bivariate Gaussian distribution at frame t, corresponding to two means, two standard deviations, and a correlation coefficient of the predicted vehicle trajectory coordinates. The means have no restrictions and do not require an activation function; the standard deviations need to ensure that they are positive, using the exponential activation function; the range of the correlation coefficient is usually between −1 and 1, which is achieved using the hyperbolic tangent activation function.

#### 2.3.3. Model Training Configuration

The model undergoes end-to-end training with a defined structure. The double-layer LSTM model is implemented in PyTorch. Training employs two loss functions: the negative log-likelihood loss for trajectory prediction and cross-entropy loss for intention prediction. The Adam optimizer updates the model parameters, which are configured with a learning rate of 0.001 and a batch size of 128. The training processes the data in batches, computes the loss, and performs backpropagation to adjust the model weights. After each epoch, the model is evaluated to monitor its performance. Finally, the trained model weights are saved for subsequent evaluation and application.

The training environment includes Python 3.10, PyTorch 2.3, and computational resources like GPUs with CUDA 11.8 and CUDNN 8700 acceleration. Key software tools such as PyTorch 2.3, CUDA 11.8, CUDNN 8700, and scipy 1.14 are utilized for deep learning development, ensuring the efficient and effective training of the model.

#### 2.3.4. Performance Evaluation Indicators

The results are reported in terms of the RMSE of the predicted trajectory with respect to the true future trajectory, over a prediction range of 0 to 5 s, as adopted in [33]. For the LSTM models generating bivariate Gaussian distributions, the means of the Gaussian components are used for RMSE calculation.

Additionally, the accuracy of predicted intentions relative to true intentions is reported. The calculation method is as follows:

(10) $$acc=\frac{\sum_{i=1}^{C}TP_{i}}{N},$$

where N represents the total number of samples, with 128 as one batch. $TP_{i}$ is the number of correct predictions in category i. C is the total number of intention category values. When calculating the lateral intention accuracy, C is taken as 3, and when calculating the longitudinal intention accuracy, C is taken as 2.

**Figure 10:**
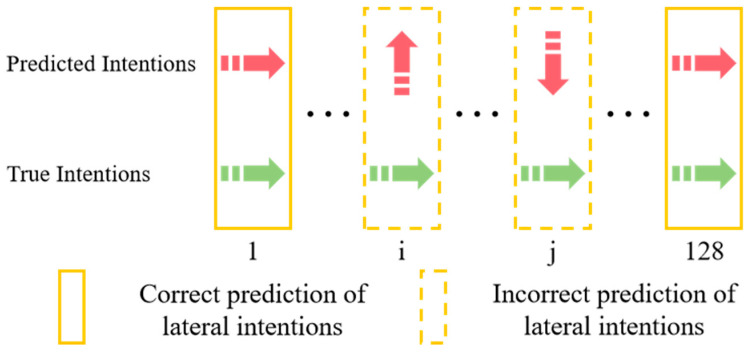
Correct and incorrect predictions of lateral intentions.Red arrow: predicted intention; green arrow: true intention. The specific lateral intentions are shown in Figure 9.

**Figure 11:**
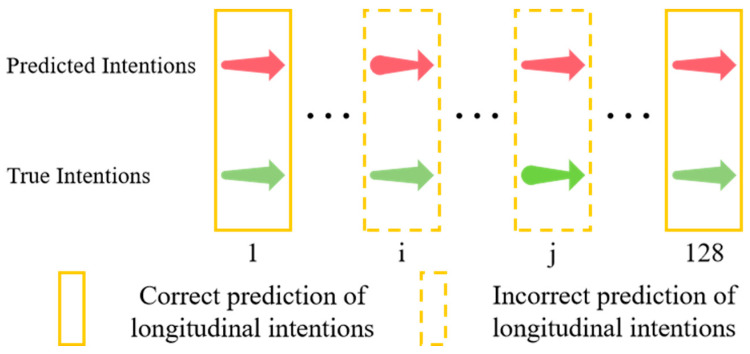
Correct and incorrect predictions of longitudinal intentions.Red arrow: predicted intention; green arrow: true intention. The specific longitudinal intentions are shown in Figure 9.

The accuracy of lateral intention and longitudinal intention are calculated separately. Figure 10 and Figure 11, respectively, illustrate the correct and incorrect predictions of lateral and longitudinal intentions.

## 3. Experiments

### 3.1. Experimental Description

In the experiments, the publicly available NGSIM US-101 and I-80 datasets [32] are utilized. These datasets contain real traffic trajectories captured by multiple sensors at a frequency of 10 Hz over a 45-min time span. The data is divided into 15-min segments, representing light, moderate, and congested traffic conditions. The coordinates of the vehicles are projected into a local two-dimensional coordinate system. The complete dataset is split into training and testing sets, with the testing set comprising 20% of the trajectories from each subset of the US-101 and I-80 datasets. The trajectories are segmented into 8-s intervals, and a 3-s trajectory data segment is used to predict a 5-s range.

To apply the model to on-vehicle camera data and integrate the prediction and perception modules, the model was tested on the track-0008 subset of the KITTI dataset [34], comprising 275 frames at a frequency of 10 Hz. Trajectories were initially tracked with camera coordinates using the 3D object tracking model and subsequently transformed into a unified world coordinate system using vehicle positioning data. The coordinate system’s point of origin is set at the primary vehicle’s initial position, with the longitudinal direction axis aligned with the heading direction of the 150th frame.

The following section compares the CS-LSTM model proposed in [22], which employs two convolutional layers and a max-pooling layer to encode the interactions of the surrounding vehicles, without considering the impact of driving style.

To further validate the superiority of our model, a comparison is also made with the recently developed MTF-LSTM model [35]. This model integrates road environment information and uses a mixed-teaching decoding method to achieve improvements in vehicle trajectory prediction accuracy and stability. However, it does not incorporate intention prediction. In this study, the mixed teaching rate is set at 0.5 for comparison.

### 3.2. Experimental Results Comparison

As one of the inputs to the model, the driving style category value is classified by the inverse cruise ratio and driving style category thresholds. Table 1 provides the proportion and trajectory counts of the three driving style categories in the NGSIM dataset, according to the thresholds mentioned above.

The model is trained in the training sets of the NGSIM dataset and is then tested in the testing sets of the NGSIM dataset. To apply the model to on-vehicle camera data and integrate the prediction and perception modules, the model is tested again in the track-0008 subset of the KITTI dataset. The comparative results are shown in Table 2 and Table 3, respectively.

Table 2 compares the RMSE values and intention accuracy rates between our model, MTF-LSTM, and CS-LSTM over a 5-s prediction horizon in the testing sets of the NGSIM dataset. The model proposed in this paper achieves lower RMSE values within the range of 0 to 5 s. Although there is little difference in the RMSE value during the first second, the RMSE reduction of 0.23 m at 5 s indicates better alignment with the actual trajectories, enhancing the safety of autonomous vehicles. In addition, the lateral intention accuracy improvement of ~1% and longitudinal improvement of ~2% enable earlier and more appropriate decision-making to avoid potential collisions. The MTF-LSTM model performs slightly better than our model in the range of 1 s to 3 s, but as time increases, our model outperforms it by 0.16 m and 0.49 m at 4 s and 5 s, respectively, demonstrating the accuracy of its long-term predictions.

Table 3 shows the comparison of RMSE values within the range of 0 to 5 s and intention accuracy rates in the track-0008 subset of the KITTI dataset. The model proposed in this paper achieves low RMSE values within the range of 0 to 2 s. In addition, the intention accuracy rates have all reached over 85%. The model proposed in this paper achieves a lower RMSE value within the range of 0 to 5 s. Although there is little difference seen in the first second, there is an improvement of 0.68 m by the fifth second, and the longitudinal intention accuracy rate is improved, with an increase of about 3%.

### 3.3. Sliding Window Length Comparison

In Reference [36], it was demonstrated that classifying the driving style based on 15 s of historical trajectory data is more reasonable. Consequently, the impact of varying sliding window lengths on trajectory prediction results is compared, as shown in Table 4, with the sliding window length adjusted from 10 to 15 s in 1-s increments.

Table 4 shows the RMSE values within the range of 0 to 5 s and intention accuracy rates with the sliding window length adjusted from 10 to 15 s in 1-s increments. It has been found that employing an appropriate sliding window length to calculate the inverse cruise ratio for driving style classification significantly improves performance compared to ignoring driving style, in terms of RMSE value and intention accuracy rates.

**Figure 12:**
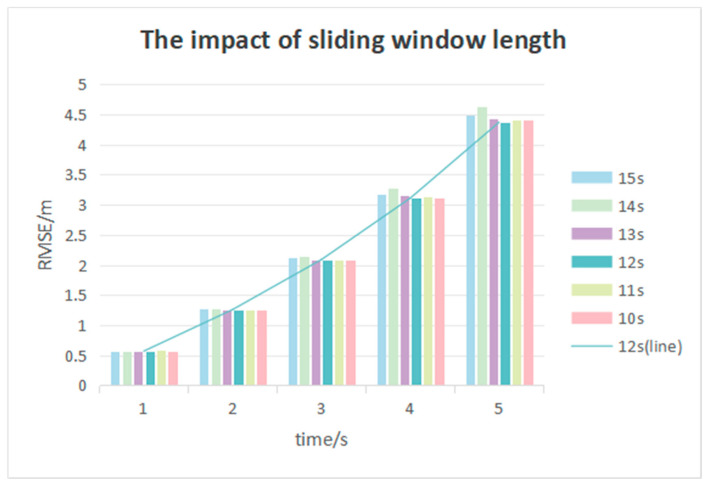
Comparison of sliding windows of different lengths.

Additionally, it has been noted in Figure 12 that employing six different sliding window lengths to calculate the inverse cruise ratio for driving style classification yields nearly identical results in terms of RMSE values within the first three seconds and intention accuracy rates. However, the RMSE values with a 12-s sliding window length being used to calculate the inverse cruise ratio for driving style classification are 0.16 m and 0.26 m lower than those with a 14-s sliding window length at the fourth and fifth seconds, respectively.

### 3.4. Grid Size Comparison in Vehicle Interaction

Table 5 shows the RMSE values within the range of 0 to 5 s and intention accuracy rates with different grid sizes during vehicle interactions. These include grid generation based on lane width and fixed size (15 feet), adaptive grid generation based on lane width and predicted vehicle length, adaptive grid generation based on predicted vehicle width and length, grid generation based on the minimum vehicle width and length of all vehicles in the dataset, grid generation based on the average vehicle width and length of all vehicles in the dataset, and grid generation based on the maximum vehicle width and length of all vehicles in the dataset.

It has been found that in the presence of lane data, adaptive grid generation based on lane width and predicted vehicle length achieves lower RMSE values within the range of 0 to 5 s than grid generation based on lane width and fixed size. Although there is not much difference seen in the first three seconds, there are improvements of 0.05 m and 0.11 m by the fourth and fifth second, respectively. In the absence of lane information, grid generation based on the maximum vehicle width and length of all vehicles in the dataset achieves the lowest RMSE values within the range of 0 to 5 s and the highest intention accuracy rates.

**Figure 13:**
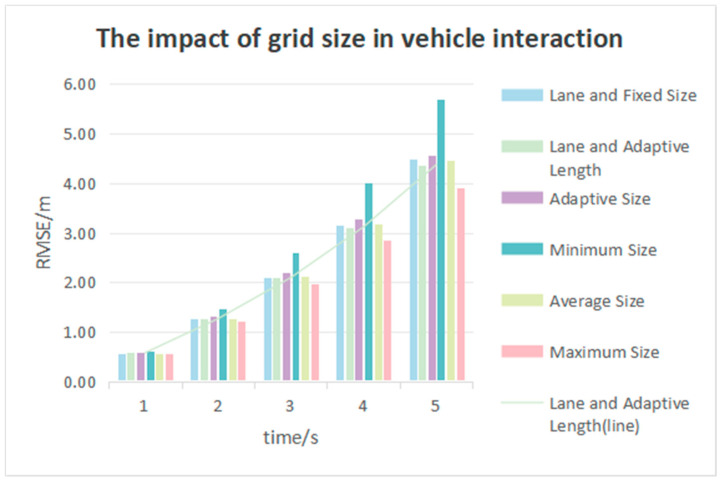
Comparison of grids in vehicle interactions according to different grid sizes.

As shown in Figure 13, the RMSE values in the first second for the different grid sizes are almost similar. It is worth noting that the RMSE value with grid generation based on the maximum vehicle width and length of all vehicles in the dataset is 1.79 m lower than that with grid generation based on the minimum vehicle width and length of all vehicles in the dataset at the fifth second.

## 4. Discussion and Conclusions

A double-layer LSTM model based on driving style and an adaptive grid is proposed for predicting target vehicle intentions and trajectory as a part of autonomous vehicle technology. This method not only utilizes trajectory coordinates sequences as the input but also integrates the category value of driving style and improves the grid generation in terms of vehicle interaction. In this way, it can better account for the vehicle’s behavioral state patterns and the interaction with surrounding vehicles. The proposed model outperforms the benchmark model on publicly available vehicle trajectory datasets in terms of RMSE values within a 5-s range and intention accuracy rates. Additionally, comparisons have been made regarding the length of the sliding window used to calculate the inverse cruise ratio for driving style classification and the size of the grid in terms of interactions with the surrounding vehicles. These comparisons verify the stability of the sliding window length used to calculate the inverse cruise ratio for driving style classification and the effectiveness of adaptive grid generation during vehicle interactions.

## Figures and Tables

**Table 1:** The proportion and trajectory count of the three driving style categories.

Driving Styles	Trajectory Counts	Proportion
Conservative	4645	39.43%
Average	4630	39.31%
Aggressive	2504	21.26%

**Table 2:** Comparison of RMSE values and intention accuracy rates in the testing sets of the NGSIM datasets.

Evaluation Metric	RMSE (m)					Lateral Intention Accuracy Rate	Longitudinal Intention Accuracy Rate
	1 s	2 s	3 s	4 s	5 s		
This Paper	0.57	1.26	2.09	3.11	4.37	98.50%	92.38%
CS-LSTM	0.59	1.29	2.15	3.24	4.60	97.75%	90.45%
MTF-LSTM	0.52	1.06	1.96	3.27	4.86	\	\

**Table 3:** Comparison of RMSE values and intention accuracy rates in the track-0008 subset of the KITTI dataset.

Evaluation Metric	RMSE (m)					Lateral Intention Accuracy Rate	Longitudinal Intention Accuracy Rate
	1 s	2 s	3 s	4 s	5 s		
This Paper	0.95	2.32	4.16	6.88	10.57	87.29%	90.52%
CS-LSTM	0.98	2.39	4.54	7.54	11.25	87.70%	87.48%

**Table 4:** Comparison of sliding windows of different lengths.

Evaluation Metric	RMSE (m)					Lateral Intention Accuracy Rate	Longitudinal Intention Accuracy Rate
	1 s	2 s	3 s	4 s	5 s		
No Style	0.59	1.29	2.15	3.24	4.60	97.75%	90.45%
15 s length	0.57	1.27	2.12	3.18	4.50	98.53%	92.18%
14 s length	0.57	1.28	2.15	3.27	4.63	98.52%	92.28%
13 s length	0.57	1.26	2.09	3.15	4.43	98.51%	92.35%
12 s length	0.57	1.26	2.09	3.11	4.37	98.50%	92.38%
11 s length	0.58	1.26	2.09	3.13	4.42	98.50%	92.56%
10 s length	0.56	1.25	2.08	3.12	4.41	98.57%	92.59%

**Table 5:** Comparison of grids of different sizes showing vehicle interaction.

Evaluation Metric	RMSE (m)					Lateral Intention Accuracy Rate	Longitudinal Intention Accuracy Rate
	1 s	2 s	3 s	4 s	5 s		
Lane and Fixed Size	0.57	1.26	2.10	3.16	4.48	98.50%	92.49%
Lane and Adaptive Length	0.57	1.26	2.09	3.11	4.37	98.50%	92.38%
Adaptive Size	0.58	1.30	2.19	3.27	4.57	98.35%	92.44%
Minimum Size	0.62	1.48	2.60	4.01	5.69	98.30%	91.20%
Average Size	0.57	1.27	2.12	3.17	4.47	98.39%	92.50%
Maximum Size	0.56	1.22	1.97	2.85	3.90	98.39%	92.80%

## Data Availability

The NGSIM vehicle trajectories open-source dataset has been used in this research. The dataset is available online and can be found here: https://ops.fhwa.dot.gov/trafficanalysistools/ngsim.htm, accessed on 12 August 2024. The KITTI dataset has also been used in this research. The dataset is available online and can be found here: https://www.cvlibs.net/datasets/kitti/eval_tracking.php, accessed on 8 December 2024.

## Abbreviations

The following abbreviations are used in this manuscript:

LSTM	Long Short-Term Memory
RMSE	Root Mean Square Error
Social-LSTM	Social-Long Short-Term Memory
AI	Artificial Intelligence
RNN	Recurrent Neural Networks
SORT	Simple Online and Real-Time Tracking
FC	Fully Connected
CS-LSTM	Convolutional Social Long Short-Term Memory
AVs	Autonomous Vehicles
NGSIM	Next Generation Simulation
MTF-LSTM	Mixed Teaching Force Long Short-Term Memory
